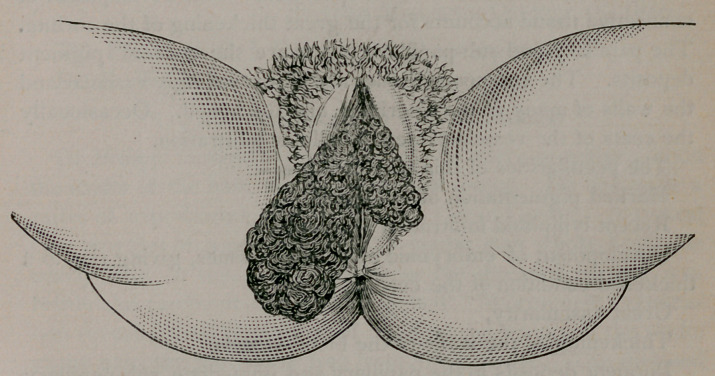# A Case of Hypertrophy of the Nymphæ

**Published:** 1886-12

**Authors:** Virgil O. Hardon

**Affiliations:** Lecturer on Operative Gynæcology, Southern Medical College, Atlanta, Ga.


### A CASE OF HYPERTROPHY OF THE NYMPHÆ.

BY VIRGIL O. HARDON, M. D.,

LECTURER ON OPERATIVE GYNÆCOLOGY, SOUTHERN MEDICAL COLLEGE, ATLANTA, GA.

S. U., colored, age 33; menstruation commenced at 14 and has always been normal. Married at 20, had one child a year, after marriage; never pregnant since. No specific history, but had gonorrhea ten years ago. Health has always been good, with the exception of recurrent attacks of pelvic inflammation, which were relieved by rest and medical treatment. (Probably exacerbations of a chronic salpingitis from the old gonorrhea.) Relief was sought for a tumor on each side of the vulva, which had appeared and grown to its present size within a year, and which interfered with locomotion, micturition and coition.

Examination showed an enlargement of both nymphæ, as shown in the accompanying cut. The surfaces were rough and rugose and the mucous membrane was converted into a thick skin, which was as deeply pigmented as the true skin, the patient being a dark mulatto. There was no pain, tenderness, ulceration or excoriation of the parts. The remainder of the vulva was perfectly normal. The clitoris was not involved in the hypertrophy. The case was regarded as one of elephantiasis of the nymphæ.

The patient was very anxious to be rid of the tumor, not because of the pain and inconvenience occasioned by it, but rather from an æsthetic point of view. The unsightly growth was an eyesore to herself and to her husband.

Accordingly, on the 9th of August, with the assistance of Dr. C. C. Greene, the patient was etherized and with scissors the nymphæ were removed *in toto*. The wound was closed with carbolized silk sutures and union took place readily by first intention.

A microscopical examination made by Dr. Henry Wile, of this city, showed that the condition was not that of elephantiasis, but of conversion of the mucous membrane into true skin with marked hypertrophy of all its structures. His report upon the specimen is as follows:

"Under a power of 75 diameters the epidermis is seen to be loosely attached to the papillary structure of the skin, and in a state of desquamation from exposure. In some places a complete separation is noticed. The cells of the rete show decided pigmentation, and this is most marked in the layer which is next to the papillæ of the corium. The papillæ of the corium present a swollen condition and are the seat of a lymphoid cell infiltration which extends downward into the deeper layer of the corium in the form of streaks, apparently in the line of the ascending capillaries. The corium is greatly thickened and is the seat of inflammatory change, which is marked by variously sized groups or aggregations of lymphoid cells, especially in the neighborhood of the blood-vessels.

Under a power of 200 diameters the layers of epidermis are seen to be in a state of desquamation. The strata are separated here and there, and in many places form only a thin covering for the subjacent structures. The lowest layers of the rete are markedly pigmented. The papillæ of the corium are enlarged and filled with a lymphoid cell infiltration. This infiltration also characterizes the other layers of the corium. In the immediate neighborhood of the blood-vessels in many places the infiltration shows evidence of recent formation by its irregular arrangement and unorganized appearance; whereas, in other places the development of these centres of infiltration into embryonic connective tissue is a notable feature of the specimen. This development of connective tissue accounts for the great thickening of the corium. The papillary and sub-papillary layers are the seat of pigment deposits. The cutaneous tissues are all exceedingly vascular and the walls of many of the arterioles are thickened. Occasionally the coats of the vessels present a cellular infiltration.

The peculiarities of this specimen are—

Marked pigmentation of epidermis,

Recent lymphoid infiltration of corium,

Development of embryonic connective tissues, giving rise to a thickened condition of the corium,

Great vascularity,

Thickening of the walls of the blood-vessels,

Pigment deposits in the papillary and immediate sub-papillary layers."

## Figures and Tables

**Figure f1:**